# Complete Genome Sequence of *Salmonella enterica* Serovar Typhimurium Strain YU15 (Sequence Type 19) Harboring the *Salmonella* Genomic Island 1 and Virulence Plasmid pSTV

**DOI:** 10.1128/genomeA.00252-16

**Published:** 2016-04-14

**Authors:** Claudia Silva, Edmundo Calva, José L. Puente, Mussaret B. Zaidi, Pablo Vinuesa

**Affiliations:** a Departamento de Microbiología Molecular, Instituto de Biotecnología, Universidad Nacional Autónoma de México, Cuernavaca, Morelos, Mexico; b Microbiology Research Laboratory, Hospital General O’Horan and Infectious Diseases Research Unit, Hospital Regional de Alta Especialidad de la Península de Yucatán, Mérida, Yucatán, Mexico; c Programa de Ingeniería Genómica, Centro de Ciencias Genómicas, Universidad Nacional Autónoma de México, Cuernavaca, Morelos, Mexico

## Abstract

The complete genome of *Salmonella enterica* subsp. *enterica* serovar Typhimurium sequence type 19 (ST19) strain YU15, isolated in Yucatán, Mexico, from a human baby stool culture, was determined using PacBio technology. The chromosome contains five intact prophages and the *Salmonella* genomic island 1 (SGI1). This strain carries the *Salmonella* virulence plasmid pSTV.

## GENOME ANNOUNCEMENT

We present the complete genome sequence of *Salmonella enterica* subsp. *enterica* serovar Typhimurium strain YU15, which was isolated in 2000 in Yucatán, Mexico, from a stool culture from a 3-month-old child with diarrhea, as part of an epidemiological surveillance program in which it was referred as YUHS 00-228 (1, 2). Multilocus sequence typing showed that this strain belongs to the sequence type 19 (ST19), which was the second most abundant in Mexico (2) and the most abundant and founder genotype for worldwide *S*. Typhimurium strains (3).

Genomic DNA was extracted by standard protocols (4) and sheared into ~10- to 20-kb fragments for PacBio library preparation and P6-C4 sequencing on one single-molecule real-time (SMRT) cell. The continuous long reads were assembled using the HGAP/Quiver-protocol in SMRT Portal version 2.3.0.140936.p4 (5), resulting in an assembly with 2 contigs. These were circularized by trimming the terminal repeats with Minimus2 (6) and subjected to four consecutive rounds of read remapping with the RS_Resequencing.1 module for sequence polishing, resulting in a final assembly with a mean coverage of ~181×. The size of the assembled genome is 5,027,649 bp, with a G+C content of 52%, comprising a 4.9-Mb chromosome and a 94-kb *Salmonella* virulence plasmid (pSTV).

Gene calling and annotation were performed with a modified version of Prokka (7). A total of 5,024 genes, including 4,725 coding sequences (CDSs) and 9 pseudogenes, were identified. Additionally, genes for 85 tRNAs, 22 rRNAs, and 1 transfer-messenger RNA (tmRNA) were annotated, plus 169 noncoding RNAs (ncRNAs), 3 clustered regularly interspaced short palindromic repeat (CRISPR) arrays, 5 riboswitches, and 446 signal peptides.

The annotation was manually curated, adding prophage predictions made by the PHAST server (8), and genomic islands detected by IslandViewer3 (9). Five complete prophages were located on the chromosome: ST104, Gifsy-2, ST64B, Gifsy-1, and a P2-like phage, plus several phage remnants. The P2-like phage was similar to prophage 3 reported in the DT104 chromosome (10). In addition to the *Salmonella* pathogenicity islands (SPIs) frequently found in *S*. Typhimurium strains, strain YU15 contains inserted in its chromosome a 43,670-bp *Salmonella* genomic island 1 (SGI1) identical to that reported for strain DT104 (11). The serovar-specific plasmid pSTV of YU15 is highly similar to those reported for other ST19 *S*. Typhimurium strains (12).

### Nucleotide sequence accession numbers.

The complete sequences of the chromosome and the pSTV of *S*. Typhimurium strain YU15 are available in GenBank under accession numbers CP014358 and CP014359, respectively.
